# Supplementary material for: The Soluble Urokinase-Type Plasminogen Activator Receptor as a Biomarker for Survival and Early Treatment Effect in Metastatic Colorectal Cancer
Source: Cancers (Basel). 2021 Oct 12;13(20):5100. doi: 10.3390/cancers13205100 (PMC8534079; doi:10.3390/cancers13205100)
Supplement: Supplementary file 1 [file cancers-13-05100-s001.zip › cancers-1375714-supplementary.pdf]

Supplementary Materials

The Soluble Urokinase-Type Plasminogen Activator Receptor as a Biomarker for Survival and Early Treatment Effect in Metastatic Colorectal Cancer

Kristian Blomberg, Torben F. Hansen, Claus L. Brasen, Jeppe B. Madsen, Lars H. Jensen and Caroline B. Thomsen

1. ROC-curves for death during suPAR study

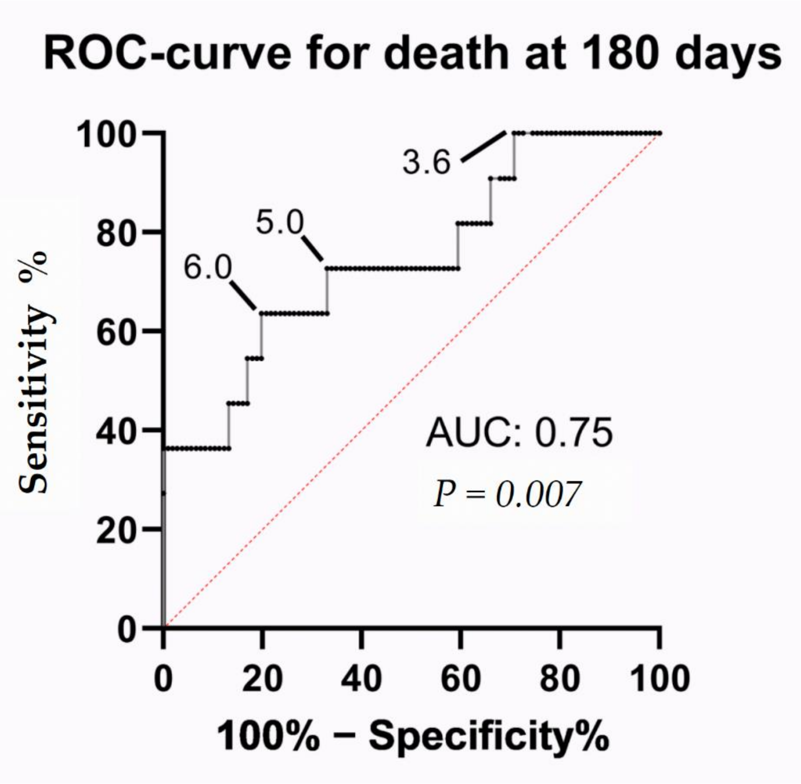

Figure S1. Receiver operating characteristics (ROC) curves of death at 180 days. The specific potentially relevant suPAR cut off threshold has been noted on each figure. Area under curve (AUC) = 0.75.

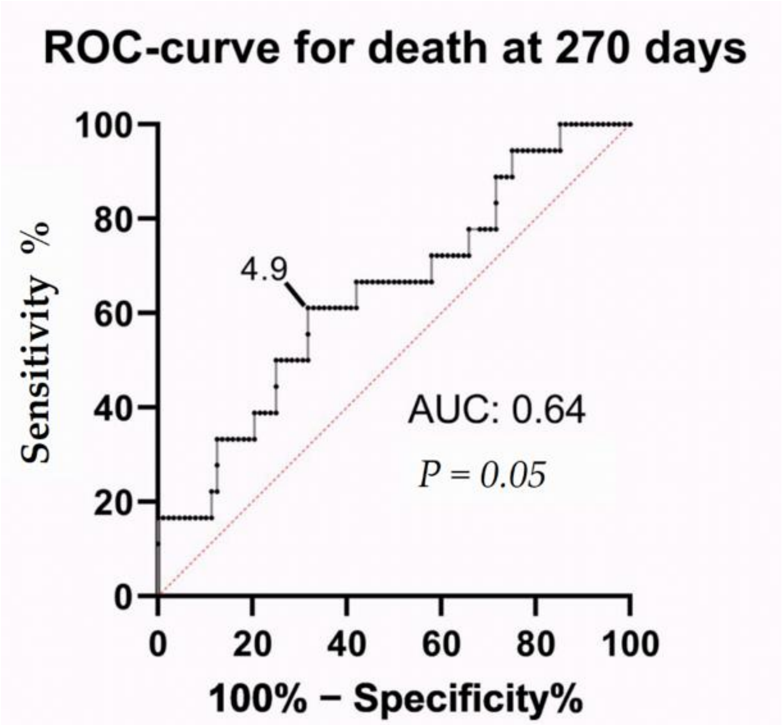

Figure S2. Receiver operating characteristics (ROC) curves of death at 270 days. The specific potentially relevant suPAR cut off threshold has been noted on each figure. Area under curve (AUC) = 0.64.

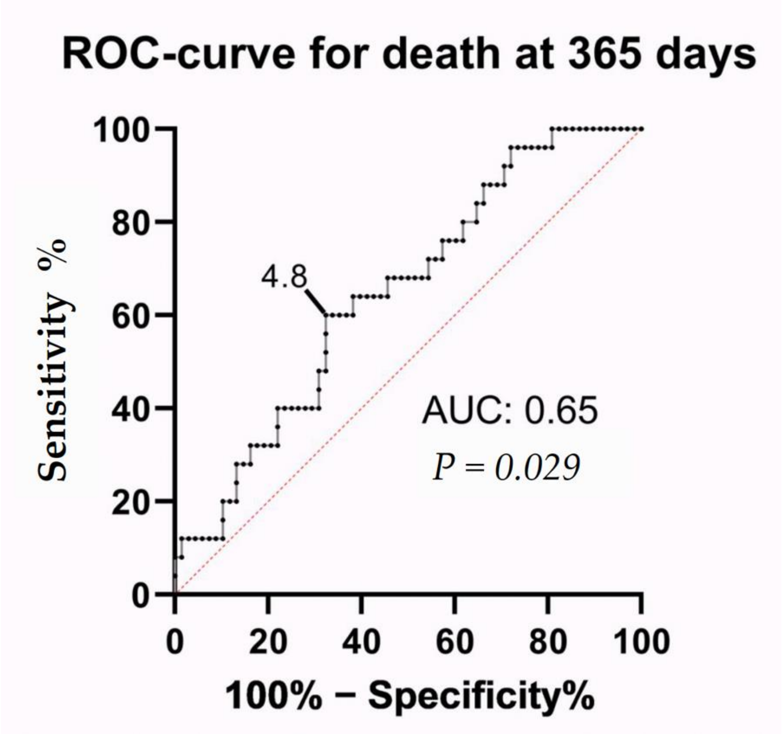

Figure S3. Receiver operating characteristics (ROC) curves of death at 365 days. The specific potentially relevant suPAR cut off threshold has been noted on each figure. Area under curve (AUC) = 0.65.

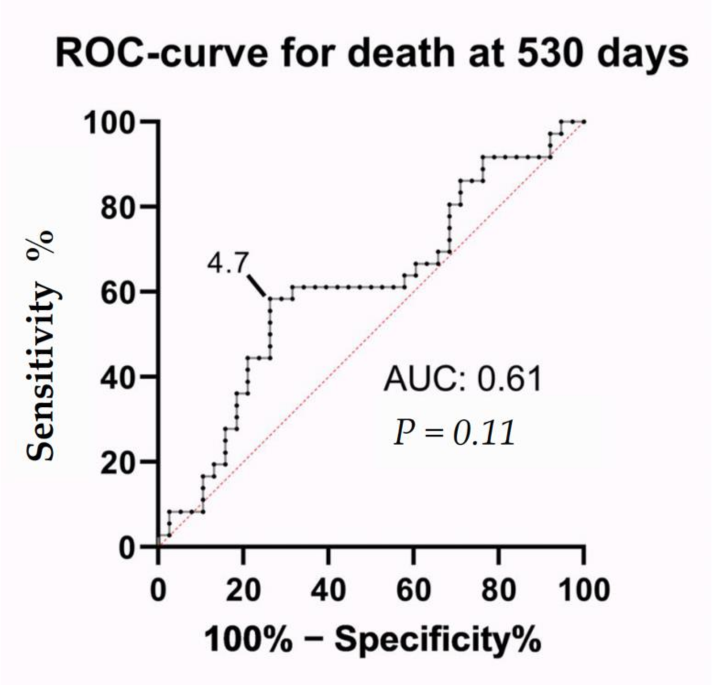

Figure S4. Receiver operating characteristics (ROC) curves of death at 530 days. The specific potentially relevant suPAR cut off threshold has been noted on each figure. Area under curve (AUC) = 0.61.

© 2021 by the authors. Licensee MDPI, Basel, Switzerland. This article is an open access article distributed under the terms and conditions of the Creative Commons Attribution (CC BY) license (http://creativecommons.org/licenses/by/4.0/).
